# MicroRNA‐containing extracellular vesicles released from endothelial colony‐forming cells modulate angiogenesis during ischaemic retinopathy

**DOI:** 10.1111/jcmm.13251

**Published:** 2017-06-20

**Authors:** Margaret Dellett, Eoin D. Brown, Jasenka Guduric‐Fuchs, Anna O'Connor, Alan W. Stitt, Reinhold J. Medina, David A. Simpson

**Affiliations:** ^1^ Centre for Experimental Medicine School of Medicine, Dentistry and Biomedical Sciences Queen's University Belfast Faculty of Medicine Health and Life Sciences The Wellcome‐Wolfson Institute Belfast Co Antrim UK

**Keywords:** microRNA, extracellular vesicle, exosome, angiogenesis, endothelial colony‐forming cell, gene expression

## Abstract

Endothelial colony‐forming cells (ECFCs) are a defined subtype of endothelial progenitors that modulate vascular repair and promote perfusion in ischaemic tissues. Their paracrine activity on resident vasculature is ill‐defined, but mediated, at least in part, by the transfer of extracellular vesicles (EVs). To evaluate the potential of isolated EVs to provide an alternative to cell‐based therapies, we first performed a physical and molecular characterization of those released by ECFCs. Their effects upon endothelial cells *in vitro* and angiogenesis *in vivo* in a model of proliferative retinopathy were assessed. The EVs expressed typical markers CD9 and CD63 and formed a heterogeneous population ranging in size from ~60 to 1500 nm by electron microscopy. ECFC EVs were taken up by endothelial cells and increased cell migration. This was reflected by microarray analyses which showed significant changes in expression of genes associated with angiogenesis. Sequencing of small RNAs in ECFCs and their EVs showed that multiple microRNAs are highly expressed and concentrated in EVs. The functional categories significantly enriched for the predicted target genes of these microRNAs included angiogenesis. Intravitreally delivered ECFC EVs were associated with the vasculature and significantly reduced the avascular area in a mouse oxygen‐induced retinopathy model. Our findings confirm the potential of isolated EVs to influence endothelial cell function and act as a therapy to modulate angiogenesis. The functions associated with the specific microRNAs detected in ECFC EVs support a role for microRNA transfer in mediating the observed effects.

## Introduction

With an ageing population and increased prevalence of diabetes and hypertension, ischaemic vascular diseases, such as peripheral artery disease 1, myocardial infarction, and ischaemic retinopathies, are a growing health issue. Timely promotion of reparative angiogenesis is an attractive therapeutic approach and preferable to blocking the later uncontrolled neovascularization which is so damaging, particularly in the retinopathies. Administration of endothelial progenitor cells with defined phenotype and vasoreparative phenotype 2, known as endothelial colony‐forming cells (ECFCs), has been demonstrated to protect against acute kidney injury 3, peripheral artery disease 1 and reduce ischaemia in animal models of hindlimb ischaemia 4 or ischaemic retinopathy 5.

Although ECFCs have been reported to incorporate into new vessels 5, their vasoregenerative effects have also been attributed to paracrine actions 3, 6 mediated, at least in part, by extracellular vesicles (EVs). Recognition of the therapeutic potential of EVs from mesenchymal and neural stem cells and cardiac and endothelial progenitor cells 7, 8 has made them the focus of intense study. They have been demonstrated to transfer proteins, mRNAs, and microRNAs to recipient cells 9, 10, 11, 12, 13, 14, 15 and have the ability to modulate endothelial cell behaviour *in vitro* 16, 17, 18, 19 and angiogenesis *in vivo* 18, 20. Translation of EV‐associated mRNAs can occur in recipient cells 9 and has been implicated in the activation of an angiogenic programme in endothelial cells 17. However, EVs carry predominantly small RNAs, of which microRNAs form a significant proportion 9, 12. We and others have demonstrated that many microRNAs are enriched within the RNA isolated from EVs relative to the originating cells, suggesting that microRNAs may be selectively incorporated into EVs 21, 22. Intercellular transfer of these microRNAs *via* EVs can regulate the gene expression 23 and function of recipient cells 10, 11, 24. Administration of ECFC exosomes protects against ischaemic acute kidney injury 3 and the microRNA content of these exosomes, specifically miR‐486‐5p, contributes to this protective effect 11.

EVs can be classified into two main types: exosomes, which are ~50–120 nm in size and released when endosomal multivesicular bodies fuse with the plasma membrane, and ectosomes (also known as microvesicles or shedding vesicles), which are generally larger (~50–1500 nm) and are formed by budding from the plasma membrane 8, 15, 25, 26, 27. In this study, we use the term ‘EVs’ to refer to the total population of vesicles isolated by ultracentrifugation. The heterogeneity of EVs, which vary in size and content between cell types, provides a challenge for the isolation of a defined product with potential as a therapeutic agent 8. We have therefore begun to characterize ECFC EVs by studying their morphology, microRNA content, uptake and effect upon endothelial gene expression.

When the blood supply to the retina is impaired, this can result in uncontrolled proliferation of new, leaky blood vessels. The resultant loss of vision is experienced in several eye diseases, including diabetic retinopathy, retinal vein occlusion and retinopathy of prematurity. Current therapeutic strategies aimed at blocking the proliferation include inhibiting VEGF; however, there are mounting concerns over the long‐term effects of chronic VEGF inhibition. If administration of EVs collected from ECFCs can promote vascular regeneration, this approach could provide a cell‐free alternative to cell‐based therapies that are hampered by low survival rates and the risk of stem cell tumorigenesis 28. We demonstrate the ability of EVs injected into the vitreous to reach the retinal vasculature and reduce the avascular area in a mouse model of proliferative retinopathy.

## Materials and methods

### Cell culture

ECFCs were isolated under full ethical approval from umbilical cord blood (~5 ml) of volunteers at the Royal Victoria Hospital, Maternity Unit, Belfast, UK. Isolation followed a protocol described previously 2, 5. Density gradient centrifugation was employed to isolate the mononuclear cell layer, which was resuspended in EGM‐2 medium supplemented with growth factors (EGM‐2 Endothelial Growth SingleQuot; Lonza, Slough, UK) with 12% foetal calf serum (FCS) and incubated on collagen‐coated plates. After 24 hrs, mononuclear cells (MNCs) were washed with EGM‐2 medium to remove any non‐adherent cells. MNCs were cultured for up to 4 weeks with media changed every 48 hrs. Cells of a cobblestone appearance with a highly proliferative nature appeared after 2–4 weeks of culture. The identity of ECFCs was confirmed by immunophenotyping for a combination of markers used to distinguish ECFCs from other mononuclear cells: endothelial markers CD‐31 and CD‐105, haematopoietic markers CD‐45 and CD‐14 and progenitor makers CD‐34 and CD‐117 (Fig. S1). From this point, all supplemented FCS was depleted of EVs by ultracentrifugation for 18 hrs at 100,000 × *g* (Type P28S rotor, HITACHI, JP‐K value 217).

Human retinal microvascular endothelial cells (h.RMECs) were obtained from Cell Systems (Kirkland, WA, USA). Cells were grown on gelatine‐coated flashes, in CSC‐complete medium (4Z0‐500; Cell Systems). Human microvascular endothelial cells (HMEC‐1) were provided by Francisco Candal 29 and were cultured in MCDB supplemented with 10% foetal bovine serum (FBS), l‐glutamine (2 mM), epidermal growth factor (10 ng/ml), hydrocortisone (1 μg/ml) and antibiotics as described previously 29.

### Isolation of EVs

Extracellular vesicles were prepared from ECFC cell culture medium by differential centrifugation. Briefly, conditioned media (containing vesicle‐depleted FCS) were harvested after 48 hrs, centrifuged at 3500 × *g* for 30 min. to eliminate cells and at 10,000 × *g* (Himac CP100WX Ultracentrifuge; Hitachi, Tokyo, Japan) in a swing bucket rotor (P28S; Hitachi) for 30 min. to eliminate cellular debris. Vesicles were pelleted by ultracentrifugation at 100,000 × *g* for 120 min. (*K* value 217). The EV pellet was resuspended in PBS and repelleted at 100,000 × *g* for 2 hrs at 4°C. The collected EVs were either stored at −80°C or stained using CM‐DiI lipid membrane stain (stock 1 mg/ml, 2.5 μl per ml of PBS) (Thermo Fisher Scientific, Waltham, MA, USA). EVs were washed twice in PBS and collected by ultracentrifugation at 100,000 × *g* for 120 min.

### Transmission Electron Microscopy

Pellets comprising EVs obtained by ultracentrifugation were prepared for TEM following collection either directly or from the resulting pellet of binding to 4 μm CD63‐coated latex beads. EVs were fixed in 2.5% glutaraldehyde in 0.1 M sodium cacodylate buffer (pH 7.4) for 1 hr. Following post‐fixation with 1% osmium tetroxide (0.1 M sodium cacodylate buffer, pH 7.4, at room temperature for 1 hr), the pellet was washed for 3 × 10 min. in 0.1 M sodium cacodylate buffer (pH 7.4). The sample was dehydrated by several washes of increasing ethanol concentration. Finally, the sample was washed twice with dry 100% ethanol for 30 min. A 1:1 ratio of dry ethanol to Agar low viscosity resin (LVR) (Agar Scientific, Essex, UK) was added. After 30 min., further Agar LVR was added to a final 1:2 ratio and left overnight under gentle movement to allow ethanol to evaporate off. The Agar LVR resin was polymerized at 60°C until the resin hardened. Ultrathin sections were obtained on a Leica Ultracut and collected on copper grids (Agar Scientific). Grids were stained with saturated alcoholic uranyl acetate and aqueous lead citrate. Stained sections were viewed with a JEOL^®^ 100CXII transmission electron microscope.

### Flow cytometry

Flow cytometry was performed according to the method of Lässer *et al*. 30. Briefly, 4‐μm latex beads (Aldehyde/Sulfate Latex Beads 4 μm; Thermo Fisher Scientific) were coated with anti‐CD63 antibody (BD Biosciences, Oxford, UK). For each sample (antibody), EVs were added at a volume equal to a minimum of 15 μg of EV protein to ~100,000 beads. The beads and EVs were incubated at 4°C overnight under gentle movement. To block, the beads were incubated with 300 μl of 200 mM glycine for 30 min. The beads were washed (PBS containing 2% FCS) at 600 × *g* for 10 min., and this was repeated for a total of two washes. EVs were then stained against antibody of choice (CD63 eBioscience 12‐0639, CD9 eBioscience 1:20); after washing, the EV–bead complexes were resuspended in 500 μl PBS and assessed using a flow cytometer (Attune^®^ Acoustic Focusing Cytometer; Thermo Fisher Scientific).

### Protein extraction

EV protein was extracted using radioimmunoprecipitation assay (RIPA) buffer supplemented with cOmplete Mini, EDTA‐free, Protease Inhibitor Cocktail (Roche, Burgess Hill, UK). Quantification was performed using a Micro BCA™ Protein Assay Reagent Kit (Thermo Fisher Scientific). The protein concentrations were used to determine the dose of EVs delivered in subsequent experiments.

### RNA extraction

Total RNA (including small RNAs) was isolated from cells and EVs using the miRNeasy mini kit according to the manufacturer's protocol (Qiagen, Crawly, UK). RNA quantity and quality were assessed using the Qubit (Thermo Fisher Scientific, UK) and Bioanalyzer (Agilent Technologies, Santa Clara, CA, USA), respectively.

### Illumina Bead Chip array

EVs were harvested from ECFC‐conditioned media after 48 hrs of incubation. EVs isolated from ECFC‐conditioned medium (T75 flask) were added to HMEC‐1 cells (T25 flask) and incubated for 48 hrs. Total RNA was extracted as above from three independent samples of untreated or EV‐treated cells and gene expression analysis was performed using the HumanHT‐12_V4 array from Illumina (San Diego, CA, USA) following the manufacturer's instructions. Gene expression data obtained from Illumina Beadstudio were normalized using ‘R’ Bioconductor with ‘lumi’ package 31. Functional analysis of genes with >1.5‐fold altered expression was performed using Ingenuity Pathway Analysis (IPA; Qiagen).

### Small RNA sequencing and analysis

Three ECFC clones were cultured and RNA extracted from the cells and EVs collected from their cell culture medium. One library was prepared from cellular RNA and one from EV RNA from each of the three clones using the Truseq small RNA Kit (Illumina), and DNA sequencing was carried out on a MiSeq in the GenePool genomics facility in the University of Edinburgh (https://genomics.ed.ac.uk/). FastQ files were trimmed and aligned to MiRBase version 21 32 (using the CLC Genomics workbench 9.0.1 (Qiagen)), allowing a maximum of two mismatches and five nucleotides shorter or longer than the mature sequence (RNA‐Seq data sets summarized in Table S1). All microRNAs both 5p and 3p represented by at least 10 reads in total across all samples were retained for further analysis. Empirical analysis of differential gene expression was performed with the ‘exact test’ for two‐group comparisons developed by Robinson and Smyth and implemented in the EdgeR package 33. MicroRNA target genes were predicted using IPA with information from TargetScan, miRecords, and TarBase 34, 35.

### Vesicle uptake studies

h.RMECs were stained with calcein (Thermo Fisher Scientific) and incubated with DiI‐stained EVs. Cells were fixed in 4% paraformaldehyde for 10 min. at room temperature, followed by two PBS washes. Cover slips were then mounted on glass slides using mounting media containing the nuclear stain DAPI (Vectashield; Vector Laboratories, Peterborough, UK) and imaged using a Nikon inverted microscope TE2000‐U with confocal unit C1 (Nikon, Surrey, UK). For quantification, five fields were selected randomly. Samples assessed using the Leica TCS SPS II were additionally stained with TO‐PRO‐3^®^ (Thermo Fisher Scientific).

### Oxygen‐induced retinopathy model

All experiments conformed to U.K. Home Office regulations (Project Licence No. 2611) and were approved by Queen's University Belfast Ethical Review Committee for Animal Research. Oxygen‐induced retinopathy (OIR) was conducted in C57/BL6J wild‐type mice according to the protocol of Smith *et al*. In this model, seven‐day‐old (P7) mouse pups and their nursing dams were exposed to 75% oxygen (humidified medical‐grade oxygen controlled by an Oxycycler C42 (BioSpherix, Parish, NY, USA)) for 5 days causing vaso‐obliteration and cessation of development of the central retinal capillary beds. On postnatal day 12 (P12), the mice were returned to room air, after which there was acute retinal ischaemia in the avascular regions of the central retina, followed by a potent pre‐retinal neovascular response between P15 and P21.

### Intravitreal injection

At P13, pups (*n* = 11) received a 1 μL intravitreal injection in the right eye (2 μg of protein content) which had been labelled with DiI‐CM (Thermo Fisher Scientific). PBS was used as vehicle and injected in the left eye of each pup as a control. All pups were killed at P17 with sodium pentobarbital, and eyes were enucleated and fixed in 4% paraformaldehyde. Retinal flat mounts were stained with isolectin B4 (Sigma‐Aldrich, Gillingham, UK) and streptavidin‐Alexa Flour488 (Thermo Fisher Scientific). Nuclei were counterstained with TO‐PRO‐3 (Thermo Fisher Scientific). Stained flat mounts were imaged with a Leica SP5 confocal microscopy. Multiple images per retina with magnification ×10 were stitched together and the neovascular and avascular areas quantified using NIS elements software (Nikon). For cell identification, antibodies (IBA1 (Wako), CD31 and F4/80 (Bio‐Rad, Watford, UK)) were used following the manufacturer's recommendations.

### Statistical analyses

To assess vesicle uptake, one‐way anova with post‐test for linear trend was performed using Prism (GraphPad, La Jolla, CA, USA). Cell migration and retinal avascular and neovascular areas were assessed using *t*‐tests performed in Prism.

## Results

### ECFC cell and EV phenotyping

Isolated ECFCs tested highly positive for endothelial markers CD‐31 and CD‐146, whereas it tested negative for haematopoietic markers CD‐45 and CD‐14 (Fig. S1). These results are comparable with previously characterized ECFC 5 and suggest that these cells possess an endothelial phenotype.

**Figure 1:**
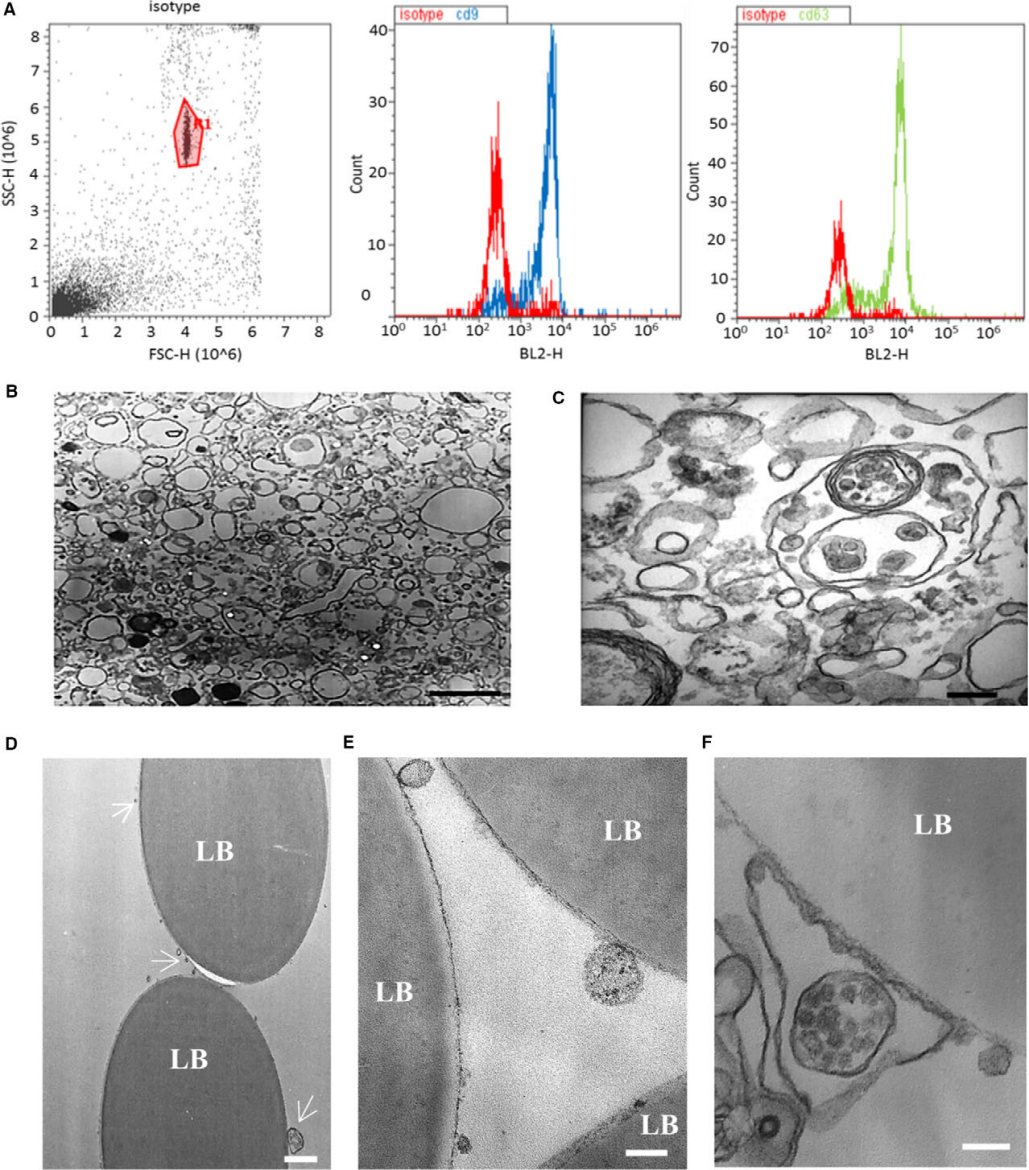
Characterization of endothelial colony‐forming cells (ECFC)‐derived extracellular vesicles (EVs). (**A**) Isolated ECFC‐derived EVs were conjugated to CD63‐coated latex beads to aid detection by flow cytometry. The gated bead‐bound population (left panel) tested positive for EV markers CD9 and CD63. (**B**) Electron micrograph demonstrating the heterogeneity of the EV population (scale bar 1 μm). (**C**) Higher‐magnification electron micrograph showing EVs of variable sizes and including a multivesicular body (scale bar 200 nm). (**D–F**) Electron micrographs of EVs bound to latex beads (LB) coated with anti‐CD63 antibody. (**D**) White arrows indicate CD63‐positive EVs of varying sizes (scale bar 1 μm). (**E**) Higher‐magnification micrograph of EVs (scale bar 200 nm). In (**F**), a multivesicular body appears to be encapsulated within a CD63‐positive membrane (scale bar 200 nm).

EVs isolated from ECFCs were first characterized by surface immunophenotyping for known vesicle markers CD9 and CD63. These tetraspanins were chosen because their expression across a broad range of tissues and abundance in vesicle membranes has made them classical markers of exosomes. However, they are also widely distributed in the plasma membrane and therefore present in other vesicles 36, with CD9 reported in large vesicles 37 and detected by flow cytometry in both microvesicles and exosomes 38. To aid in the detection of EVs by flow cytometry, they were bound to aldehyde/sulphate latex beads, which had been pre‐coated with anti‐CD63 antibody to enable adhesion of CD63‐positive EVs. These EV–bead complexes were then immunostained for CD9 and CD63. Flow cytometric analysis of ECFC‐derived EV–bead complexes showed a distinct population of beads with an average overall signal from bound EVs that were positive for these EV markers (Fig. 1A).

### ECFCs release a diverse population of EVs

To assess the size range and morphology of isolated ECFC‐derived EVs, TEM was performed. This revealed an extremely diverse and complex population of EVs, varying in both morphology and diameter (Fig. 1B–F). Ranging in size from 60 to 120 nm in diameter with electron‐dense interiors, many of the EVs are consistent with characteristics reported for exosomes 12, 15, 27. A small number of EVs with more electron‐dense structures and a diameter of approximately 60 nm may be a subpopulation of exosomes. The more numerous larger EVs have diameters of 140–1500 nm and lack an electron‐dense interior characteristics of ectosomes (microvesicles) 12, 15.

A fourth population of EVs was associated with more than one membrane. Some appeared as a large electron lucid vesicle containing one or more smaller electron‐dense vesicles, similar to the multivesicular bodies reported to be released from other cell types 39. There were rare occurrences of a single vesicle encapsulated within many layers of membranes, similar to those reported in human blood 40, ejaculates 41 and mouse lymph 42. TEM was used to investigate the morphology of the CD63‐positive EVs bound to anti‐CD63‐coated aldehyde/sulphate latex beads detected by flow cytometry. As with the previous TEM analysis of all the EVs present in the pellet collected by ultracentrifugation, a diverse population of EVs were observed bound to CD63‐coated beads. The CD63^+^ EVs displayed a similar range of sizes and morphologies (Fig. 1D–F). This included a multivesicular body encapsulated within an outer membrane (Fig. 1F).

### Interaction and uptake of ECFC EVs by h.RMECs

**Figure 2:**
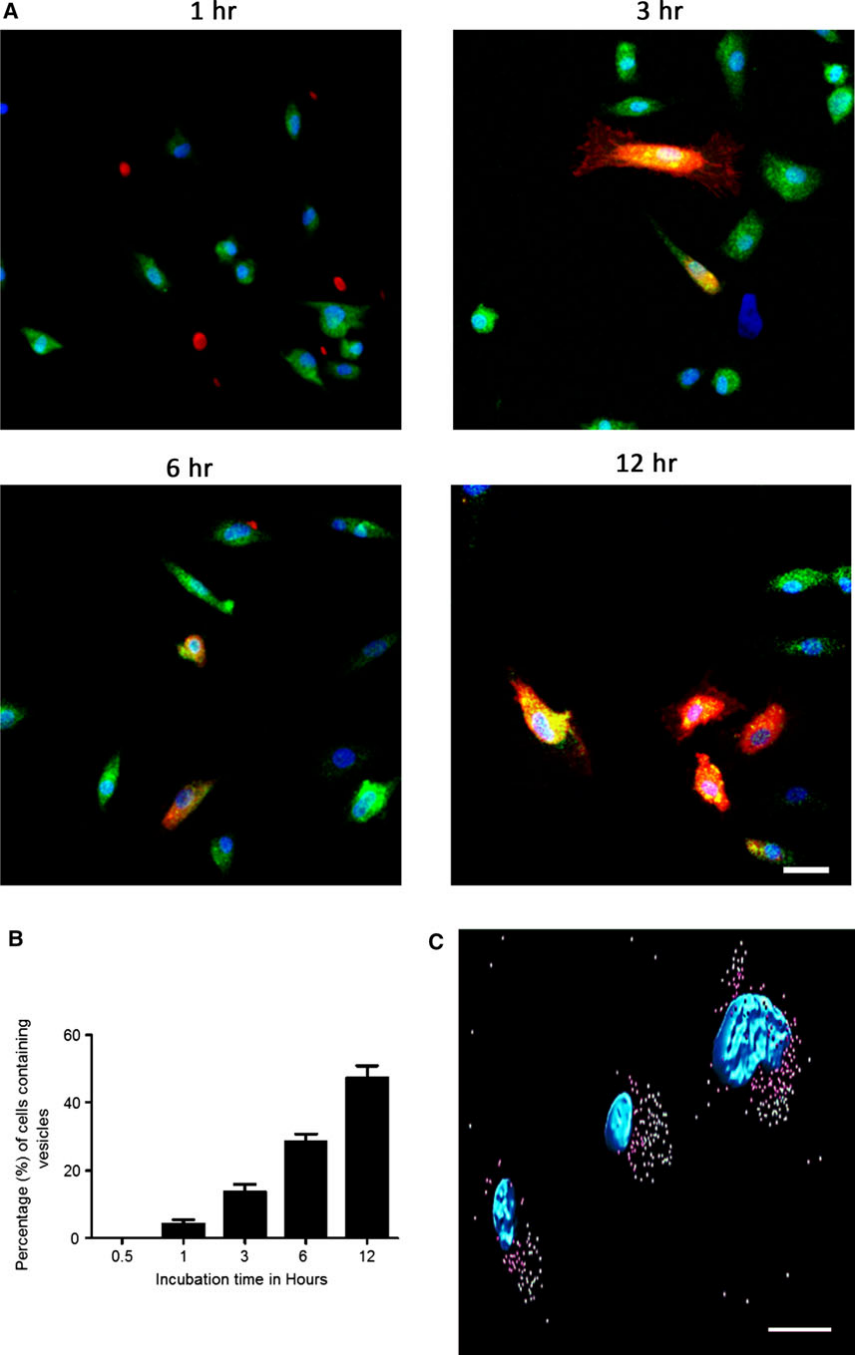
Uptake of labelled endothelial colony‐forming cells (ECFC) extracellular vesicles (EVs) by hRMECs. (**A**) ECFC EVs labelled with DiI (red) were incubated for 1, 3, 6 or 12 hrs with h.RMECs labelled with calcein (green cytoplasm) and DAPI (blue nuclei). Colocalization of red and green dyes indicates that the vesicles were internalized by specific individual cells, whereas other cells remained unlabelled. (**B**) The number of cells containing EVs increased with time. There was a linear increase in the number of cells labelled with DiI as incubation times increased (*P* < 0.0001, anova with post‐test for linear trend) and the cells also appeared more brightly labelled. This is a representative experiment showing the mean from *n* = 5 fields for each time‐point (error bars represent standard error). (**C**) Internalized ECFC EVs (surface rendered pink near to nuclei, green further away) were frequently observed localized to one side of the recipient h.RMEC nucleus (blue) (scale bar 10 μM).

To assess the potential for ECFC‐derived EVs to influence angiogenesis, their interactions with human retinal microvascular endothelial cells (h.RMEC), a key target cell type, were investigated. Confocal microscopy imaging of h.RMECs after incubation with EVs labelled with DiI confirmed their cellular uptake (Fig. 2A). Some cells appeared to contain large quantities of EVs, whereas others had no detectable internalized EVs. The number of h.RMECs taking up EFCF EVs was not dose dependent, but the fluorescence intensity of positive cells increased with dose of labelled EVs (data not shown). EVs were observed as both small and large aggregated clumps within the perinuclear region, as reported previously 43, and were often located primarily to one side of the nucleus (Fig. 2C, Fig. S2). To determine the effect of time of exposure to EVs upon uptake, the number of cells containing EVs was measured at several time‐points over a 12‐hrs period. Pre‐stained ECFC EVs were added at T0 and imaged at time‐points up to 12 hrs. No EVs were detected in cells prior to one hour of incubation; thereafter, the number of cells containing EVs was calculated as a percentage. The percentage of cells that had taken up EVs increased with time, suggesting that a subset of the h.RMEC cell population is receptive to EFCF EV uptake in a time‐dependent manner. Cells also became brighter in the red channel (DiI‐stained EVs) with time, presumably due to additional vesicles being taken up by receptive cells (similarly, incubation with higher concentrations of EVs also increased cell fluorescence (data not shown)).

### ECFC EVs promote h.RMEC migration

**Figure 3:**
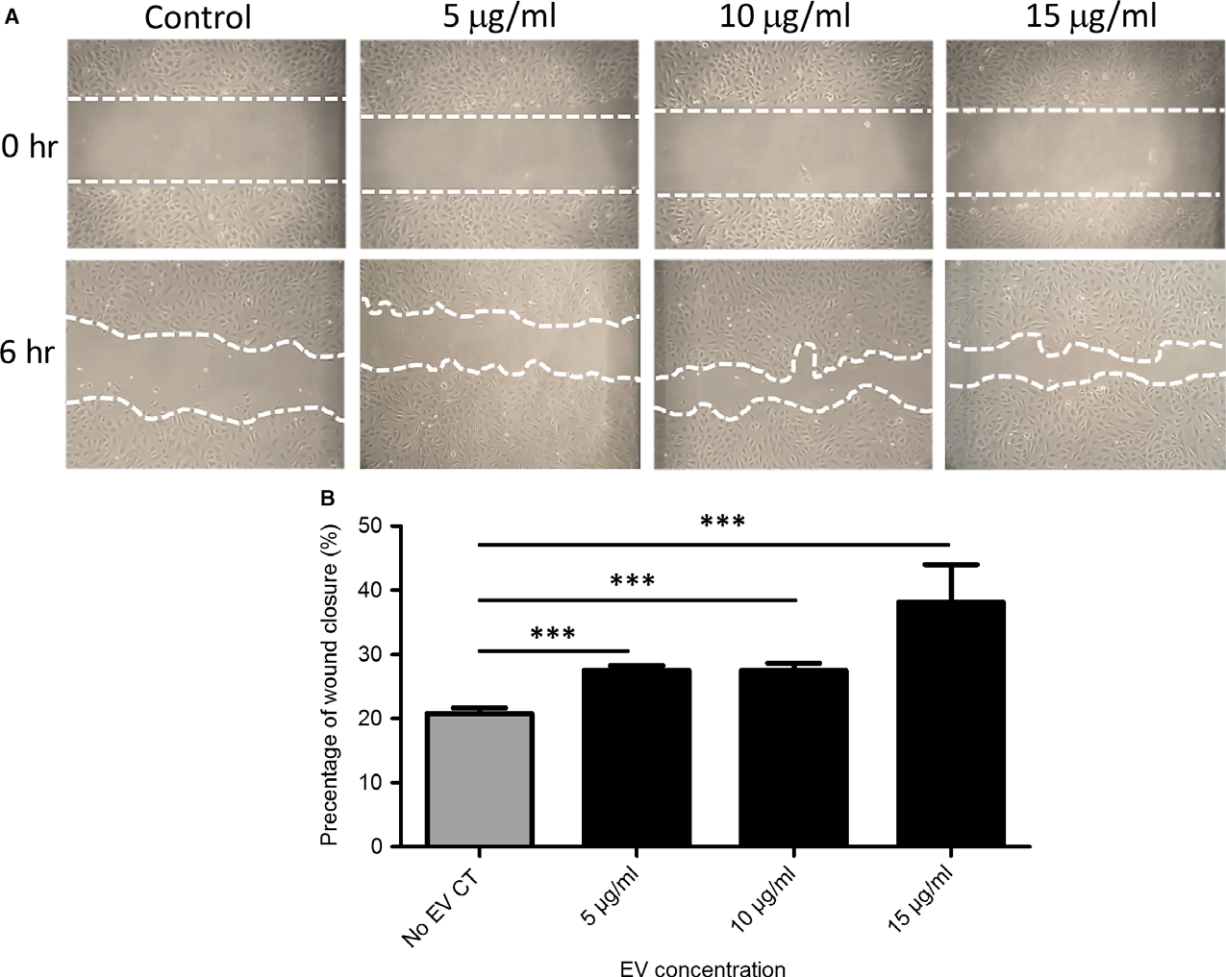
Endothelial colony‐forming cells (ECFC)‐derived extracellular vesicles (EVs) promote migration of h.RMECs. (**A**) Representative images of scratch wound assays to assess the effect of ECFC EVs (5, 10, 15 μg/ml and no EV control) upon h.RMEC migration. (**B**) A significant difference (***P ≤ 0.05) was observed between the no EV control and each ECFC‐derived EV treatment (*n* = 3, error bars represent standard error).

A scratch was generated in a monolayer of confluent h.RMECs as described previously 44, and the effect of ECFC‐derived EVs was assessed. The effect of three concentrations of ECFC‐derived EVs (5, 10 and 15 μg/ml) upon wound closure was assessed in comparison with no EV controls. There was a significant difference (*P* ≤ 0.05) observed between percentage of wound closure after 6 hrs in the no EVs control (20%) and 5 μg/ml (28%), 10 μg/ml (27.5%) and 15 μg/ml (38%) treatments (Fig. 3). This suggests that ECFC‐derived EVs can confer a pro‐migratory potential to h.RMECs.

### Influence of ECFC EVs upon endothelial gene expression

Global changes in gene expression caused by incubation of human microvascular endothelial cells with ECFC EVs for 48 hrs were measured by microarray. Expression of 674 genes was altered with a fold change of ≥1.5 in either direction (*P* < 0.05), with 284 increased and 390 reduced. The ten most altered genes in either direction are shown in Table 1, and the complete list is provided in Data S1. Several genes amongst those most increased are involved in extracellular matrix (ECM) remodelling, matrix metalloproteinase 1 (MMP1), which is involved in the degradation of ECM, and fibulin 2 (FBLN2). The heparan sulphate‐modifying enzyme SULF1, which mediates heparan sulphate–VEGFA interactions and inhibits angiogenesis 45, was down‐regulated by EVs. Also down‐regulated are ACVRL1—mutations in which cause vessel malformations in hereditary haemorrhagic telangiectasia 46—endothelin 1 (EDN1), which encodes vasoconstrictive peptides, and CCL2, which has chemotactic activity for monocytes and basophils.

**Table 1:** Most altered genes in endothelial cells treated with endothelial colony‐forming cells extracellular vesicles (EVs) as determined by microarray

Gene symbol^a^	Mean control	Mean EV‐treated	Fold change
SERPINB2	2.63411	3.3392	5.07
TPM2	2.875	3.5333	4.553
FOXD1	2.94311	3.5516	4.059
MMP1	2.9084	3.5055	3.954
SERPINB2	2.4888	3.0631	3.752
MIR1974	2.7794	3.3509	3.728
TMEM200A	2.63601	3.1849	3.539
DSE	2.5991	3.1376	3.455
TMEM154	2.6664	3.1985	3.404
TPM2	3.3014	3.8096	3.222
MIR1978	3.30111	3.7876	3.065
FBLN2	2.8572	3.3401	3.04
ACVRL1	3.1429	2.6265	−3.283
CD34	3.09659	2.5676	−3.38
EDN1	3.20119	2.6508	−3.551
BGN	3.9473	3.3693	−3.784
CCL2	3.4507	2.8375	−4.103
AIF1L	3.7038	3.0686	−4.317
SULF1	3.0162	2.364	−4.489
CRYAB	3.403	2.7049	−4.989
ITM2A	3.06829	2.3437	−5.303
ALDH1A1	3.4686	2.5408	−8.468
ALDH1A1	3.53339	2.5101	−10.55

a Genes appearing more than once are represented by independent probes in the microarray.

The most significantly enriched functional categories amongst those genes with altered expression (>1.5) are listed in Table 2, the two most significant being ‘development of vasculature’ and ‘angiogenesis’. Of those categories for which it was possible to predict a direction of effect based on prior knowledge of the causal effects of the genes involved, the most decreased (activation *z*‐score <−2) involved in migration of myeloid cells (Data S2), whereas the most activated involved in cell proliferation and cancer.

**Table 2:** Significantly altered functional categories

Categories	Diseases or functions annotation	*P*‐value
Cardiovascular system development and function	Development of vasculature	6.12E‐26
Cardiovascular system development and function, organismal development	Angiogenesis	3.17E‐24
Cellular growth and proliferation	Proliferation of cells	2.37E‐22
Cardiovascular system development and function, organismal development	Vasculogenesis	2.34E‐21
Cellular movement	Cell movement	5.86E‐19
Cell death and survival	Necrosis	1.04E‐18
Tissue development	Growth of epithelial tissue	2.82E‐18
Cardiovascular system development and function, cellular development, cellular function and maintenance, cellular growth and proliferation, organismal development, tissue development	Endothelial cell development	3.41E‐18
Cell death and survival	Apoptosis	8.34E‐18
Cellular movement	Migration of cells	2.80E‐17
Cardiovascular system development and function, cellular development, cellular function andmaintenance, cellular growth and proliferation, organismal development, tissue development	Proliferation of endothelial cells	1.10E‐16
Cardiovascular system development and function, cellular movement	Cell movement of endothelial cells	2.66E‐16
Cell death and survival	Cell death	2.90E‐16
Tissue development	Development of epithelial tissue	7.83E‐16
Cardiovascular system development and function, cellular movement	Migration of endothelial cells	8.11E‐15

**Figure 4:**
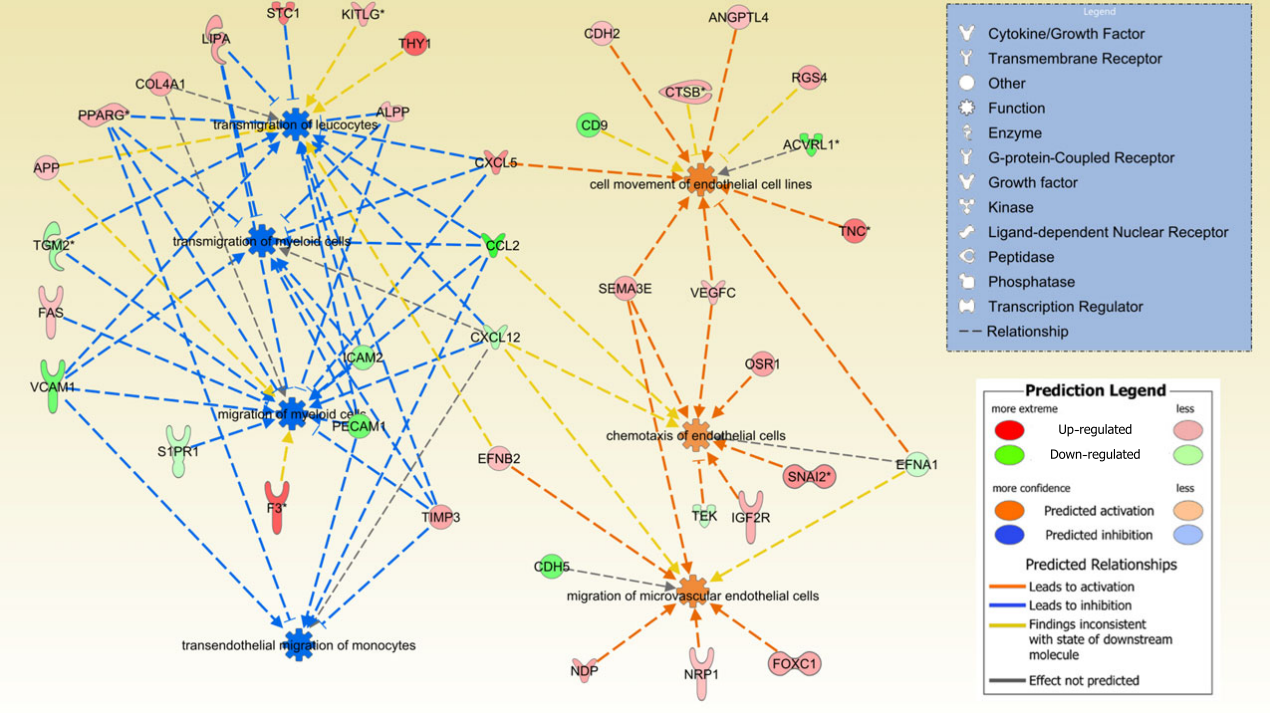
Addition of extracellular vesicles (EVs) is predicted to alter the activity of functional categories associated with ‘cellular movement’. For functional categories significantly enriched in altered genes, as detected by microarray following treatment of hRMECs with EVs, both the direction of change and predicted effect of each gene were considered. Migration and transmigration of myeloid cells, transendothelial migration of monocytes and transmigration of leucocytes were predicted to be decreased in activity (activation *z*‐score <−2). The six categories most likely to be enhanced in activity (activation *z*‐score 1‐1.5) included migration of microvascular endothelial cells, cell movement of endothelial cell lines and endothelial cell.

When considering functional categories associated with ‘cellular movement’, although migration and transmigration of myeloid cells, transendothelial migration of monocytes and transmigration of leucocytes were significantly enriched in altered genes and predicted to be decreased in activity (activation *z*‐score <−2), the six categories most likely to be enhanced in activity (activation *z*‐score 1–1.5) included migration of microvascular endothelial cells, cell movement of endothelial cell lines and endothelial cell chemotaxis (Fig. 4).

### MicroRNA content of ECFC vesicles

**Figure 5:**
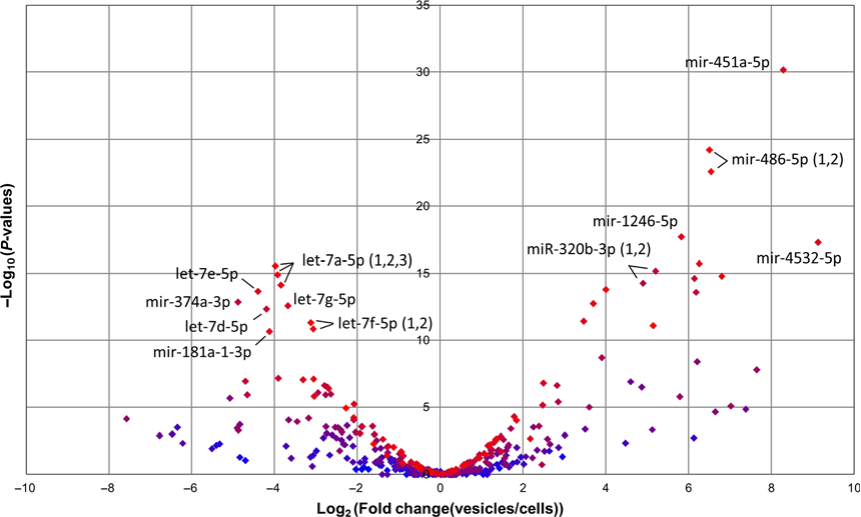
MicroRNAs differentially expressed between cells and vesicles. The volcano plot highlights all microRNAs differentially expressed between endothelial colony‐forming cells (ECFC) cells and vesicles (Bonferroni‐corrected *P* values <0.05). The colour of each spot indicates the expression level (blue low, red high) of the microRNA that they represent. The identities of the most significantly different and highly exported (*e.g*. miR‐451 and miR‐486‐5p) or retained (members of the let7 family) microRNAs are labelled.

Small RNA sequencing was used to identify the profiles of microRNAs present within ECFCs and exported in their vesicles. The 15 most highly expressed microRNAs identified in cells and EVs are listed in Table 3 (all microRNAs listed in Data S3). Those moderate to highly expressed microRNAs (mean normalized expression combined between cells and vesicle >200 reads) differentially expressed between cells and vesicles (Bonferroni‐corrected *P* values <0.01) are shown in Table 4. A volcano plot highlighting all microRNAs differentially expressed between the compartments (Bonferroni‐corrected *P* values <0.05, Fig. 5) shows that many of the most highly exported (*e.g*. miR‐451 and miR‐486‐5p) and retained (members of the Let7 family) microRNAs concur with previous findings for ECFCs 11 and other cell types 21. The microRNA expression profiles of independent samples of EVs are more similar to one another than the profiles from a specific ECFC clone and its EVs (Fig. S3), suggesting that a conserved subset of microRNAs is exported.

**Table 3:** MicroRNAs identified in cells and extracellular vesicles (EVs) ranked by expression

MicroRNA	Cells—Normalized mean counts	MicroRNA	EVs—Normalized mean counts
mir‐10b‐5p	280,851	mir‐10b‐5p	334,402
mir‐21‐5p	192,847	mir‐30a‐5p	94,312
mir‐30a‐5p	94,476	mir‐21‐5p	45,939
mir‐10a‐5p	46,992	mir‐22‐3p	41,936
mir‐126‐5p	23,631	mir‐486‐2‐5p	36,424
mir‐22‐3p	23,134	mir‐486‐1‐5p	32,501
let‐7i‐5p	19,915	mir‐151a‐3p	29,859
mir‐151a‐3p	16,003	mir‐126‐5p	24,546
mir‐92a‐1‐3p	15,570	mir‐10a‐5p	22,513
mir‐92a‐2‐3p	14,682	mir‐25‐3p	18,841
mir‐222‐3p	13,653	let‐7i‐5p	15,672
mir‐21‐3p	13,438	mir‐221‐3p	15,626
let‐7f‐2‐5p	11,289	mir‐216a‐5p	14,126
let‐7f‐1‐5p	10,951	mir‐92a‐1‐3p	12,689
mir‐28‐3p	9673	mir‐92a‐2‐3p	12,614

**Table 4:** MicroRNAs differentially expressed >fivefold between cells and vesicles with total normalized expression >500 counts

MicroRNA	Cells—Normalized mean counts	EVs—Normalized mean counts	Fold change (Vesicles/Cells)^a^	Bonferroni p value
miR‐4532‐5p	1	1555	561.9	2.0E‐15
miR‐451a‐5p	4	2298	313.0	2.7E‐28
miR‐7704‐5p	28	4077	111.8	6.5E‐13
miR‐486‐2‐5p	284	36,424	93.4	1.0E‐20
miR‐486‐1‐5p	263	32,501	90.9	2.4E‐22
miR‐4792‐5p	40	3291	76.7	7.5E‐14
miR‐4516‐5p	5	689	72.6	1.0E‐11
miR‐3960‐3p	6	757	70.7	9.5E‐13
miR‐1246‐5p	36	3068	56.7	7.6E‐16
miR‐320b‐1‐3p	10	633	36.9	2.6E‐13
miR‐216a‐5p	333	14,126	35.5	3.1E‐09
miR‐320b‐2‐3p	13	593	29.9	2.1E‐12
miR‐143‐3p	306	6615	16.1	6.4E‐12
miR‐380‐3p	28	472	15.0	7.7E‐07
miR‐107‐3p	515	8223	13.0	7.0E‐11
miR‐432‐5p	173	2372	11.1	1.5E‐09
miR‐323a‐3p	95	843	7.1	9.4E‐05
miR‐134‐5p	327	2241	5.6	6.3E‐05
miR‐660‐5p	233	1456	5.6	2.8E‐03
miR‐20a‐5p	613	79	−6.2	4.1E‐04
miR‐151a‐5p	2166	505	−6.5	1.6E‐04
miR‐30c‐2‐5p	581	109	−6.8	4.8E‐04
miR‐26b‐5p	814	106	−6.9	9.4E‐05
miR‐30c‐1‐5p	597	104	−7.7	3.3E‐04
miR‐26a‐2‐5p	2531	524	−8.2	6.2E‐04
miR‐31‐5p	4069	540	−8.3	3.0E‐05
let‐7f‐1‐5p	10,951	1689	−8.4	5.5E‐09
let‐7f‐2‐5p	11,289	1644	−8.7	1.9E‐09
miR‐26a‐1‐5p	2552	433	−9.9	3.5E‐05
let‐7g‐5p	2710	239	−12.8	1.0E‐10
let‐7a‐1‐5p	7768	772	−14.4	3.0E‐12
miR‐1260a‐5p	882	111	−15.0	2.8E‐05
let‐7a‐2‐5p	7738	715	−15.2	4.9E‐13
let‐7a‐3‐5p	7755	677	−15.8	1.1E‐13
miR‐181a‐1‐3p	4929	348	−17.4	8.6E‐09
let‐7d‐5p	990	41	−18.3	1.8E‐10
let‐7e‐5p	2673	118	−21.1	8.6E‐12
miR‐27a‐5p	589	20	−25.3	4.8E‐04
miR‐100‐3p	726	25	−26.0	4.6E‐05
miR‐374a‐3p	833	22	−29.4	5.5E‐11

EVs, extracellular vesicles.

a Fold change from EDGE test (tagwise dispersions).

MicroRNAs work combinatorially to regulate gene expression 47, and indeed, many genes are targeted by multiple microRNAs. The genes predicted to be targeted by those microRNAs most highly expressed in EVs (top 15 microRNAs comprising ~75% of all reads, Table 3) are most significantly enriched in the functional category ‘metastasis’, with ‘development of vasculature’, ‘angiogenesis’ and ‘cell movement of endothelial cells’ also within the top 10 categories (Data S4). The microRNAs enriched in EVs (>threefold, >100 reads) have an overlapping list of predicted target genes that are significantly enriched in diseases or functions associated with cancer. To try and identify the most likely functional interactions, the predicted targets were compared with those genes down‐regulated in endothelial cells following treatment with EVs (Data S1). This identified 16 genes (listed in Data S5), which pathway analysis showed to be enriched in functions associated with ‘cardiovascular system development and function’. Notable individual genes include CDH5 (VE‐cadherin), reduction of which causes endothelial cells to adopt a migratory phenotype 48, and IGFBP5 that has been linked to endothelial cellular senescence 49.

### ECFC EVs contribute to vascular repair in the retina

**Figure 6:**
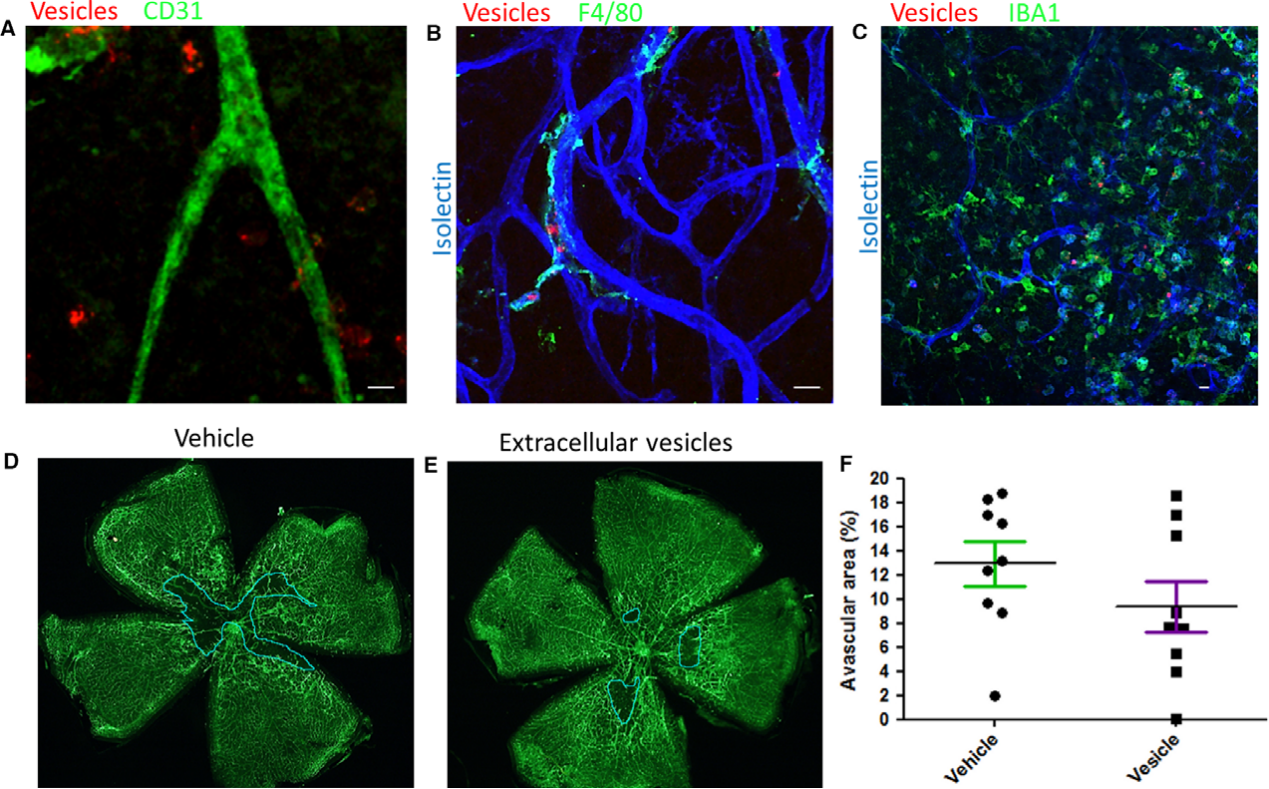
Endothelial colony‐forming cells (ECFC) extracellular vesicles (EVs) target perivascular cells and reduce avascular area in an oxygen‐induced retinopathy (OIR) model. (**A‐C**) ECFC EVs labelled with DiI (red) were injected into the vitreous of 13‐day‐old (P13) mouse pups following exposure to high oxygen. The pups were culled during the subsequent neovascular response at P17 and retinal flat mounts prepared. Red EVs were visible close to the vasculature visualized with endothelial marker CD31 (green) (**A**). Use of microglia/macrophage markers F4/80 (**B**) or Iba1 (**C**) showed EVs associated with perivascular cells (scale bar 10 μm). (**D**,** E**) To assess avascular area, retinal flat mounts prepared from eyes injected with vehicle control or EVs were stained with isolectin (green) to identify the vasculature. The central avascular area is delineated with a blue line. (**F**) There was a significant decrease in the avascular area in retinas injected with ECFC EVs (*P* ≤ 0.05) compared with vehicle (*n* = 9 in each group).

EVs injected into the vitreous were detectable in the retina of the OIR model of diabetic retinopathy. The EVs were associated with endothelial cells and perivascular cells, including some that stained positive for macrophage and microglial markers F4/80 and IBA1 (Fig. 6, Fig. S4). A significant decrease (*P* ≤ 0.05) in the avascular area at P17 was observed in mice injected with EVs *versus* vehicle control at P13 (Fig. 6), although the decrease in neovascular area was not significant (Fig. S5).

## Discussion

The molecular and functional assays reported in this study confirm the potential of ECFC EVs to modulate endothelial cell behaviour and influence angiogenesis *in vivo*, supporting previous reports that exosomes from ECFCs can block human umbilical vein endothelial cell apoptosis and protect against acute kidney injury 3. It therefore seems plausible that EVs could be used as the basis of a cell‐free therapy for ischaemic diseases that could provide many of the benefits already demonstrated for direct application of ECFC cells 8. In addition to removing any risk of neoplastic transformation, the potential advantages of this strategy include the ability to genetically engineer the contents and employ the diverse EV constituents to target multiple mechanisms simultaneously 8.

However, the diversity of EV populations presents a challenge for defining appropriate subsets for therapeutic use. The electron microscopy in this study confirmed that the diversity, both in size and in morphology, of the vesicles released by ECFCs is similar to that reported from many other cell types and observed in biofluids 12, 41, 50. This is exemplified by the observation of vesicles comprising several membranes, similar to those reported elsewhere 39, 50. The close interactions observed between the membranes of vesicles >1 μm and anti‐CD63 beads suggest that CD63 is not exclusively an exosome marker and support reports of its association with additional EV populations 51. A prerequisite for clinical applications will be development of robust protocols for the isolation of better defined EV populations.

Fluorescently labelled EVs were taken up by endothelial cells over a period of hours and localized in the perinuclear region 43, 52. Although over 50% of cells internalized EVs *in vitro*, a much smaller subpopulation was positive in the retina. It is encouraging that EVs were detectable in regions of ischaemia/angiogenesis following intravitreal delivery. This is an advantage to minimize potentially deleterious side effects from systemic delivery. ECFCs have been manipulated to improve homing to appropriate cells, for example, by overexpression of integrin β1 1, and a similar strategy could be adopted for EVs.

The observation from small RNA sequencing data that certain microRNAs are enriched in EVs relative to ECFC cells (more extremely than in the reverse direction) is consistent with preferential loading of specific microRNAs. Although the functions of most of these EV‐enriched microRNAs are unknown, several have been implicated in vascular function. Exosome‐derived miR‐486‐5p targets the phosphatase and tensin homolog (PTEN) in endothelial cells and reduces ischaemic kidney injury 11. Conversely, miR‐26a, which is retained within ECFC cells, inhibits EC migration and angiogenesis 53. Although the mechanism by which specific microRNAs are loaded into EVs is not well understood, it is possible to manipulate the microRNA content of EVs by modulating expression in the parental cells 21. This approach has already been reported for let7c in mesenchymal stem cells 54 and miR‐486‐5p in ECFC EVs 11. The small RNA sequencing data presented here provide further candidate microRNAs for development of this strategy to enhance the therapeutic properties of EVs.

The molecular characterization of ECFC EVs and demonstration of their efficacy in an *in vivo* model provide support for their development as a therapy for retinal ischaemic disease.

## Conflicts of interest

The authors confirm that there are no conflicts of interest.

## Supporting information


**Fig. S1.** Cell surface immunophenotype of ECFCs determined by flow cytometry.

**Fig. S2.** Internalization of ECFC EVs by hRMEC cells.

**Fig. S3.** Scatterplots of ECFC cellular *versus* EV microRNA expression.

**Fig. S4.** Perivascular location of intravitreally injected EVs.

**Fig. S5.** Retinal neovascular area in the OIR model for eyes injected with EVs or vehicle control.

**Table S1.** Summary of RNA sequencing data sets.

**Data S1.** Full list of significantly altered genes (fold change >1.5) in endothelial cells treated with ECFC EVs as determined by microarray.

**Data S**2. Full list of significantly enriched functional categories associated with genes altered by treatment with ECFC EVs.

**Data S3.** Full list of microRNAs detected in cells and EVs by RNA‐Seq.

**Data S4.** Full list of significantly enriched functional categories associated with target genes of microRNAs highly expressed within EVs.

**Data S5.** Gene symbol.
