# Supplementary figures and images for: MicroRNA‐containing extracellular vesicles released from endothelial colony‐forming cells modulate angiogenesis during ischaemic retinopathy
Source: J Cell Mol Med. 2017 Jun 20;21(12):3405–19. doi: 10.1111/jcmm.13251 (PMC5706503; doi:10.1111/jcmm.13251)

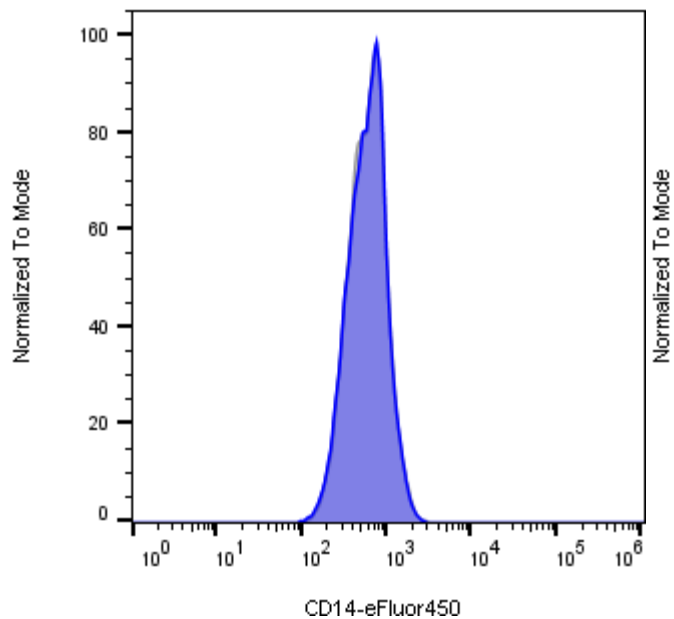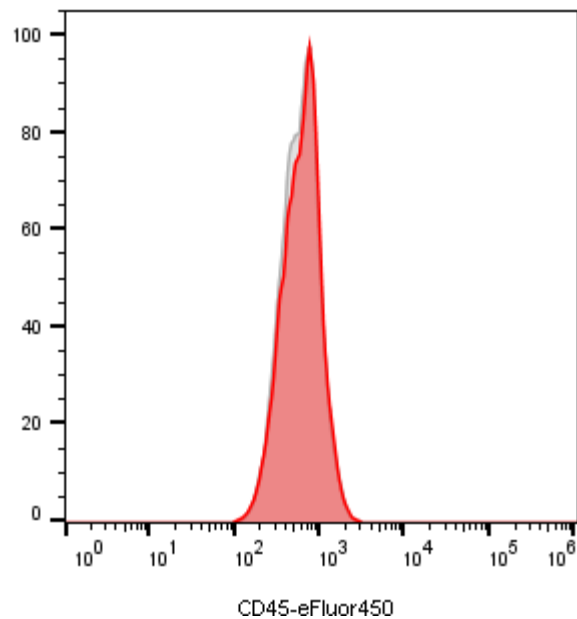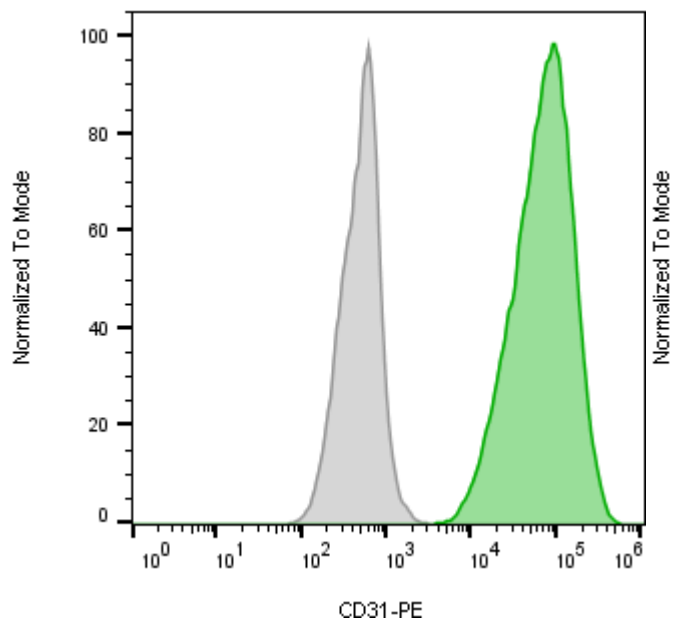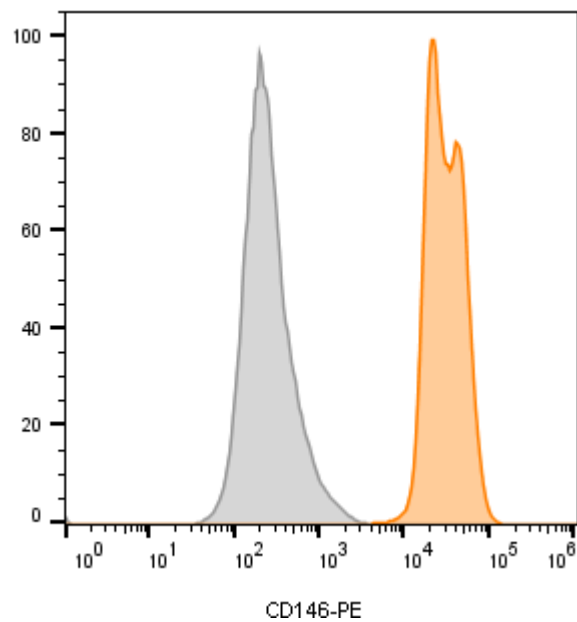

| Cell Surface Marker | Expression |
|---------------------|------------|
| CD14-eFluor450      | 0%         |
| CD45-eFluor450      | 0%         |
| CD31-PE             | 99.9%      |
| CD146-PE            | 99.9%      |

Supplement: Supplementary file 1 — Fig. S1. Cell surface immunophenotype of ECFCs determined by flow cytometry. [file JCMM-21-3405-s001.pdf]

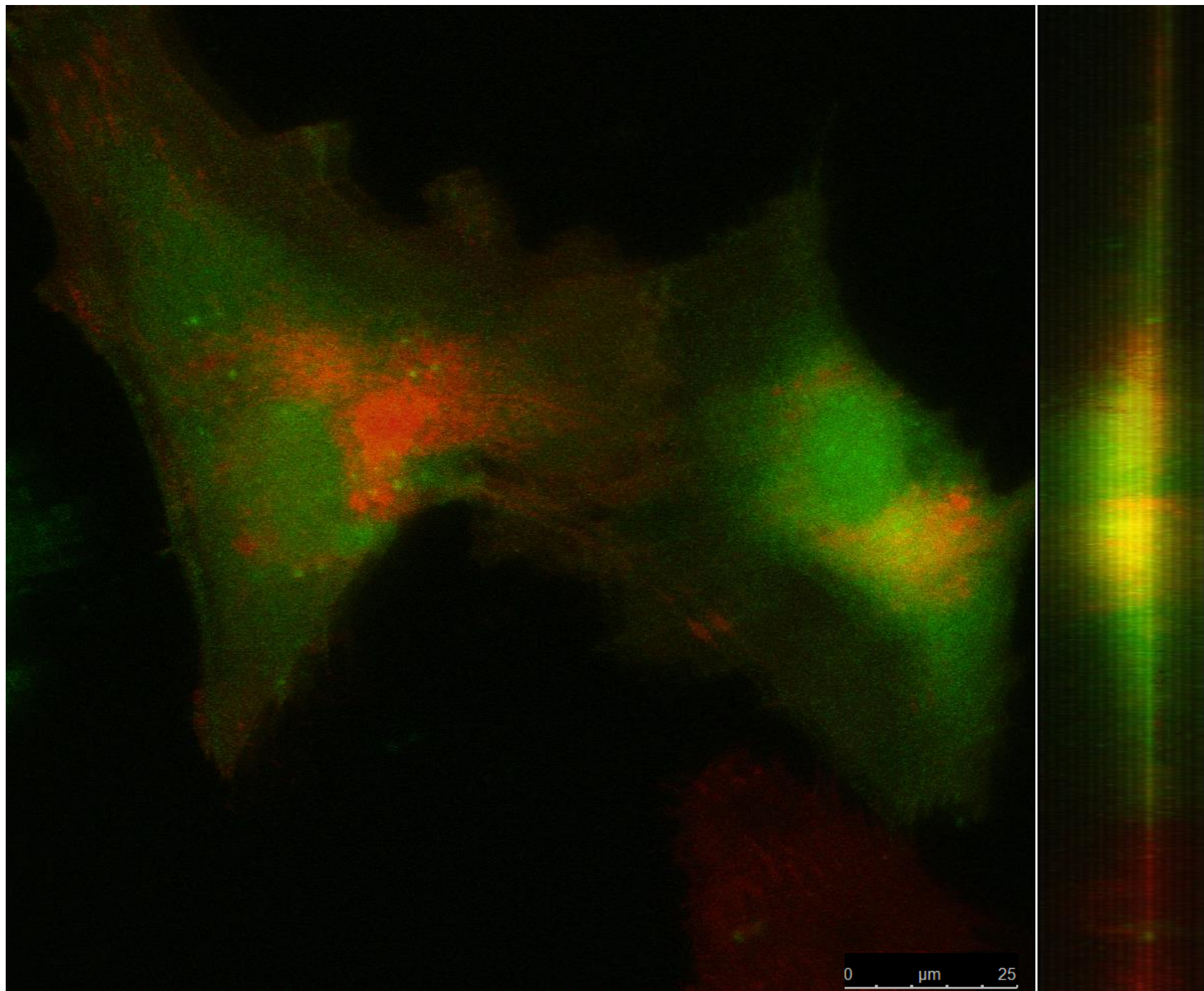

Supplement: Supplementary file 2 — Fig. S2. Internalization of ECFC EVs by hRMEC cells. [file JCMM-21-3405-s002.pdf]

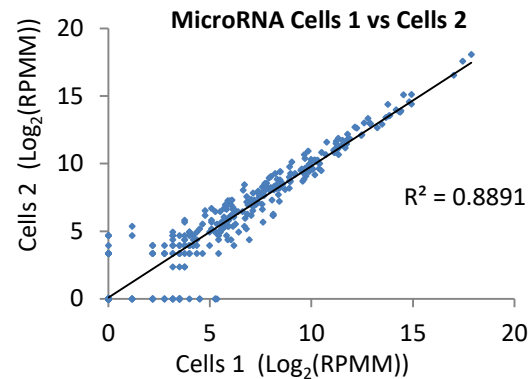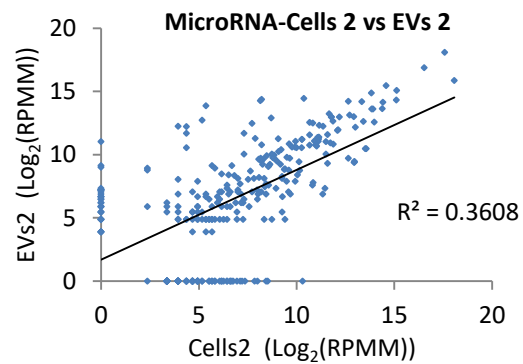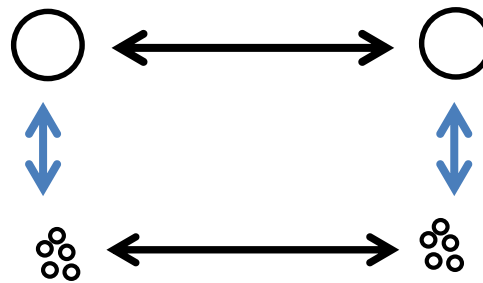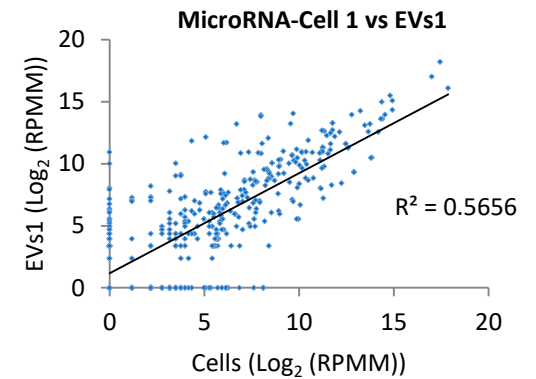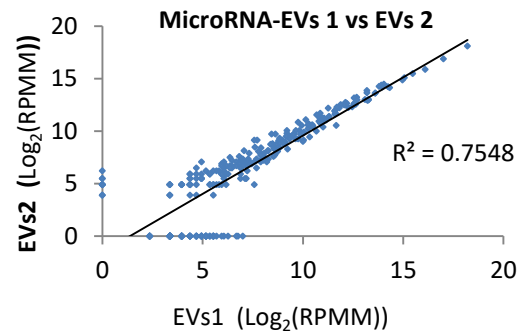

Supplement: Supplementary file 3 — Fig. S3. Scatterplots of ECFC cellular versus EV microRNA expression. [file JCMM-21-3405-s003.pdf]

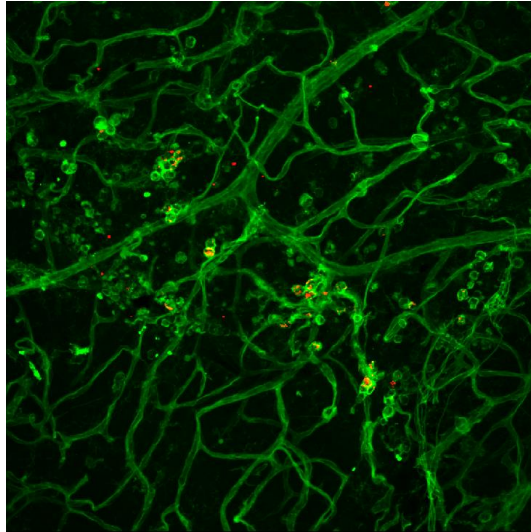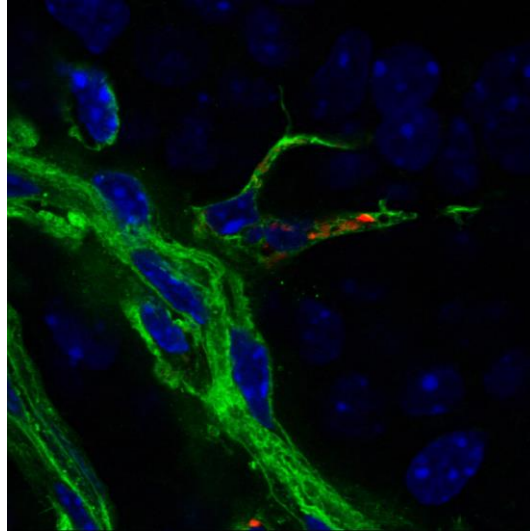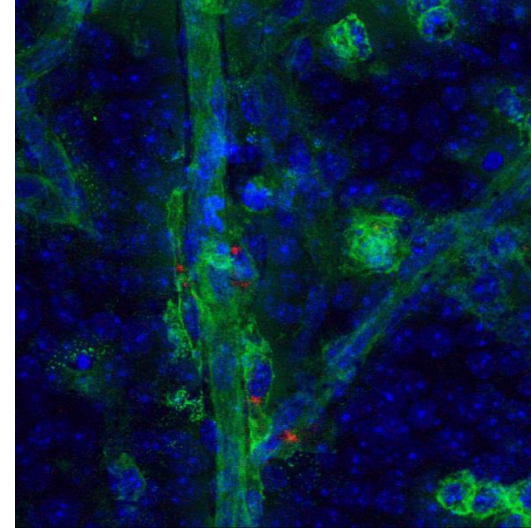

Isolectin B4  
TO-PRO-3  
DiI (EVs)

Supplement: Supplementary file 4 — Fig. S4. Perivascular location of intravitreally injected EVs. [file JCMM-21-3405-s004.pdf]

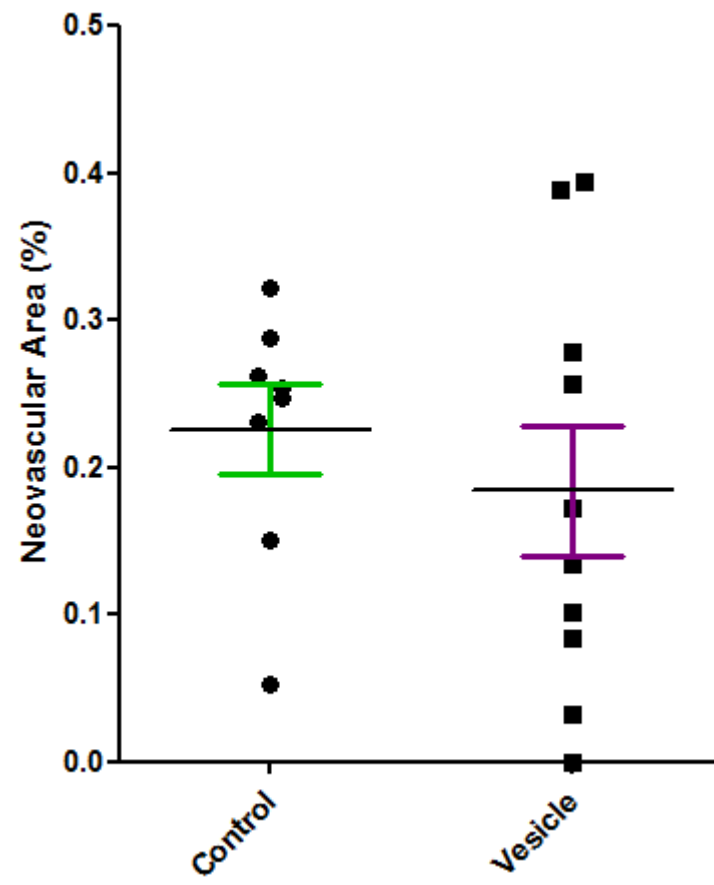

Supplement: Supplementary file 5 — Fig. S5. Retinal neovascular area in the OIR model for eyes injected with EVs or vehicle control. [file JCMM-21-3405-s005.pdf]
